# Study of Essential Oil Isolated from Achiote (*Bixa orellana*) Leaves: Chemical Composition, Enantiomeric Distribution and Antimicrobial, Antioxidant and Anticholinesterase Activities

**DOI:** 10.3390/antibiotics12040710

**Published:** 2023-04-05

**Authors:** Eduardo Valarezo, Silvia Torres-Torres, Nohely Pineda-Guarnizo, Ximena Jaramillo-Fierro, Luis Cartuche, Vladimir Morocho, Miguel Angel Meneses

**Affiliations:** Departamento de Química, Universidad Técnica Particular de Loja, Loja 110150, Ecuador

**Keywords:** biological activity, *Bixa orellana*, chiral compounds, *Enterococcus faecium*, essential oil, germacrene D

## Abstract

*Bixa orellana* is a native and cultivated species of Ecuador commonly known as achiote (annatto), this species is very versatile with a wide variety of uses and applications of its leaves, fruits and seeds. In this study, the chemical composition, enantiomeric distribution and biological activity of essential oil isolated from the leaves of *Bixa orellana* were determined. Hydrodistillation was used to isolate the essential oil. Gas chromatography coupled with mass spectrometry was used to determine the qualitative composition, a gas chromatograph equipped with a flame ionization detector was used to determine quantitative composition and gas chromatography on an enantioselective column was used to determine enantiomeric distribution. Antibacterial activity was determined using the broth microdilution method, for which we used three Gram-positive cocci bacteria, a Gram-positive bacilli bacterium and three Gram-negative bacilli bacteria. 2,2′-azinobis-3-ethylbenzothiazoline-6-sulfonic (ABTS) acid radical cation and 2,2-diphenyl-1-picrylhydryl (DPPH) free radical were used as reagents for determining the antioxidant activity of the essential oil. The spectrophotometric method was used to analyze acetylcholinesterase inhibitory effect of the essential oil. The yield of leaves in essential oil was 0.13 ± 0.01% (*v*/*w*). A total of 56 chemical compounds, which represent 99.25% of the total composition, were identified in the essential oil. Sesquiterpene hydrocarbons were the most representative group in number of compounds and relative abundance with 31 compounds and 69.06%, respectively. The principal constituents were found to germacrene D (17.87 ± 1.20%), bicyclogermacrene (14.27 ± 0.97%), caryophyllene < (E)– > (8.56 ± 1.24%) and pinene <α-> (6.34 ± 0.13%). Six pairs of enantiomers were identified in the essential oil of *Bixa orellana*. The essential oil presented strong activity against the *Enterococcus faecium* (ATCC 27270) with an MIC of 250 μg/mL and weak activity against *Enterococcus faecalis* (ATCC 19433) and *Staphylococcus aureus* (ATCC 25923) with an MIC of 1000 μg/mL. The antioxidant activity of the essential oil was strong according to ABTS methods with a SC_50_ of 61.49 ± 0.04 µg/mL and was moderate in DPPH with a SC_50_ of 224.24 ± 6,4 µg/mL. Additionally, the essential oil reported moderate anticholinesterase activity with an IC_50_ of 39.45 ± 1.06 µg/mL.

## 1. Introduction

Medicinal plants have been used for thousands of years throughout the world as a form of traditional medicine, and are an important source of active ingredients for the modern pharmaceutical industry [1]. Bixaceae are a family of various medicinal plants found in some tropical and subtropical countries, including Ecuador, Brazil, Colombia, Peru, Mexico and other Latin American countries. This family includes about 15 genera and more than 200 species. Some of the genera included in the Bixaceae family are *Cochlospermum*, *Ochroma*, *Heliocarpus*, *Ellipanthus*, *Dicella*, *Bixopsis*, *Gyrocarpus* and *Bixa* [2,3].

Ecuador is a tropical country in which the Bixaceae family is present with three genera. The genus *Amoreuxia* Moc. and Sessé ex DC. is represented by the species *Amoreuxia wrightii* A. Gray, the genus *Cochlospermum* Kunth is represented by the species *Cochlospermum vitifolium* (Willd.) Spreng., and finally, the genus *Bixa* L. is represented by the species, *B. arborea* Huber, *B. platycarpa* Ruiz and Pav. Ex G Don, *B. urucurana* Willd. and *B. orellana* L. [4].

*Bixa orellana* species is of great cultural and economic importance in several countries of the tropical and subtropical world. This species, whether native or cultivated, grows in Ecuadorian territory at an altitude of 0–1500 m.a.s.l., and occurs in the form of a shrub or small tree with a height of 3 to 5 m, sometimes reaching a maximum height of 10 m [5]. *Bixa Orellana* presents pink or white flowers, and alternate dark green leaves with extended petioles [6]. The plant species is found in the four Ecuadorian regions Galapagos, Coastal, Andean and Amazon, mainly in the provinces of Bolivar, Carchi, Chimborazo, Cotopaxi, El Oro, Galápagos, Guayas, Riobamba, Morona Santiago, Napo, Pastaza, Pichincha and Santo Domingo de los Tsáchilas. Some of the common names that local people have assigned to this species are achiote, achiote colorado, achiote de monte, achiote de racimo, Bandenu tape, duchichiimu puka, kutu chuinu puka, lala’ fintsumu mu, mu, muja, achiwiti, aya manturu, manturu, puka manturu, cu’a cuña, inszia cuña, tsanda cuña, huihue posa, muju posa, payo posa, posa, sëño posa, kaka, kakamo, kakawe, ipiák, ipiak, color, mora and annatto (English language), among others [7].

*Bixa orellana* is a very versatile species, with a wide variety of uses and applications. The leaves, fruits and seeds of the species have been reported to be edible. The yellow-orange natural dye isolated from its seeds which exhibits high biodegradability, low toxicity and compatibility with the environment, is applied in food, textile, leather, cosmetic, solar cells and other industries [8]. The aril of the seeds is used as a seasoning and to give color to food, for which it is dried and fried in oil. The leaves are also utilized as a seasoning. The fruit is used as food for birds and the stem is employed to make torches. The plant is utilized to shade crops. Alternatively, the leaves, fruit and roots are employed as personal adornment. In fact, the red pigment extracted from the stem and the seeds is used by certain indigenous groups (such as the Tsa’chi) to paint their bodies, mainly their faces and hair, in the belief that they will not be recognized by evil spirits. Shamans apply the pigment to paint their faces as a mask, which protects them from evil spirits during exorcism, they also utilize it to decorate their bodies at parties or important ceremonies, or as an aid in hunting. The red pigment is also used as a dye to paint or dye fibers, clothing, blowguns, spears, arrows and pottery [4].

The traditional medicinal use of *B. orellana* is widely spread among the Ecuadorian inhabitants. The stem, macerated in water or stem sap, is used to treat conjunctivitis. The root is applied to aid in digestion. The pigment that is extracted from the seeds is used to eliminate pimples, spots and cure skin infections caused by fungi. The seeds are also employed to relieve headaches and as an expectorant. The fruit is exploited to treat epilepsy and conditions of the prostate. From the leaves and tender flowers, a liquid is extracted for the treatment of cataracts. The flower is employed to treat heart problems. The leaves are used to treat colds, coughs, skin inflammation and to relieve muscle, bone, kidney and bladder pain. The hot leaves are applied to treat wounds and also as an ointment to relieve rheumatism. The leaves, in infusion and together with *Heliocarpus americanus*, are utilized to speed up childbirth. The decoction of the leaves is used by Kichwa women, in drinks and baths, to regain strength after childbirth and to cleanse the body. Likewise, the bath with the decoction of the leaves is applied to relieve rheumatism. The root, leaves and seeds in different preparations are used to treat mythical diseases [4].

The species *B. orellana* is rich in chemical compounds with various beneficial properties for human health. Evidence from several studies indicates that the extracts obtained from different parts of the plant present a diversity of compounds with antibacterial [9], antifungal [10], antiparasitic and insect repellent [11], antileishmanial [12], antioxidant [13], anticancer and cytotoxic [14], anti-inflammatory [15], anticlastogenic [16] and antidiarrheal [17] properties, among others. *Bixa orellana* seeds present fat-soluble carotenoid pigments, such as bixin and norbixin, which have antioxidant and anti-inflammatory properties and are widely used in the food industry as natural colorants [18]. The presence of essential oil in the leaves and seeds from *B. orellana* has been reported previously [12,19,20]. *Bixa orellana* seeds contain essential oils rich in volatile organic compounds, which are mainly terpene and sesquiterpene in nature [12,21]. Likewise, volatile compounds have been detected in the aqueous and organic extracts of *B. orellana* seeds [22]. Despite the applications in traditional medicine and common and industrial uses of the species *B. orellana*, to the best of our knowledge, not enough information has been found in the literature on the biological activity of the essential oil of *Bixa orellana* leaves. That is why the aim of this study is to determine the chemical composition, enantiomeric distribution and antimicrobial, antioxidant and anticholinesterase activities of the essential oil isolated from leaves of this species, in order to determine its potential in pharmacological applications.

## 2. Results

### 2.1. Essential Oil Isolated

Approximately 25 kg (24,930 g divided into three distillations of 6130 g, 9290 g and 9510 g) of fresh (with a moisture of 64 ± 1% *w*/*w*) *B. orellana* leaves, were hydrodistilled in a Clevenger type apparatus in order to isolate the essential oil (EO). The amount of EO obtained was approximately 32 mL, which represents a yield of 0.13 ± 0.01% (*v*/*w*) or 1.3 ± 0.1 mL/kg.

### 2.2. Physical Properties of Essential Oil

The EO from *B. orellana* presented as an unctuous liquid. Table 1 shows the mean values and standard deviations (SD) of the physical properties of essential oil. In general, the essential oil of *B. orellana* was a yellow liquid less dense than water.

### 2.3. Chemical Composition of Essential Oil

The qualitative identification of the *B. orellana* compounds was carried out by gas chromatography coupled to mass spectrometry (GC-MS) and the quantification of their relative abundances was performed by means of gas chromatography equipped with the flame ionization detector (GC-FID). The information on the compounds and their relative abundances (%) are shown in Table 2. A total of 56 chemical compounds were identified in the EO of leaves from *B. orellana*, which represent 99.25% of the total composition. The compounds were classified into four groups: monoterpene hydrocarbons (MH), sesquiterpene hydrocarbons (SH), oxygenated sesquiterpene (OS) and diterpene hydrocarbons (DH). In terms of number of compounds and relative abundance, SH was the most representative group with 31 compounds and 69.06%, respectively. Only one compound (bifloratriene) of the DH group was identified and no oxygenated monoterpene compounds or non-terpene compounds were found. The principal constituents (> 5%) were found to be SH germacrene D (CN: 25, CF: C_15_H_24_, MM: 204.19 Da) at 17.87 ± 1.20%, bicyclogermacrene (CN: 30) at 14.27 ± 0.97%, caryophyllene < (E)– > (CN: 15) at 8.56 ± 1.24%, and MH pinene < α– > (CN: 1, CF: C_10_H_16_, MM: 136.13) at 6.34 ± 0.13%.

### 2.4. Enantiomeric Analysis

Using a column with enantioselective stationary phase it was possible to separate six pairs of enantiomers from EO of *B. orellana* leaves, although it was not possible to establish the order of elution between the enantiomers (+) and (-) in four of them (α-copaene, α-trans-bergamotene, bicyclogermacrene and spathulenol). Table 3 shows the retention time (RT), enantiomers, retention indices (RI), enantiomeric distribution (ED) and enantiomeric excess (e.e.), for each pair of compounds. The (-)-α-pinene was found practically pure with e.e. of 99.63%.

### 2.5. Antimicrobial Activity

Microdilution broth method was used to determine the antibacterial activities of EO of leaves from *B. orellana*. Table 4 shows the tested microorganisms and minimum inhibitory concentration (MIC) values of EO and positive control, in addition, the values of the negative control. Ampicillin was used as a positive control for *Enterococcus faecalis*, *Enterococcus faecium* and *Staphylococcus aureus,* and ciprofloxacin for *Listeria monocytogenes*, *Escherichia coli*, *Pseudomonas aeruginosa* and *Salmonella enterica.* Dimethyl sulfoxide at 5% was used as a negative control. *Bixa orellana* EO reported MIC values of 250 µg/mL against Gram-positive cocci *Enterococcus faecium* and MIC values of 1000 µg/mL against *Enterococcus faecalis and Staphylococcus aureus.*

### 2.6. Antioxidant Activity

The antioxidant activity of essential oil from *B. orellana* was determined using the DPPH and ABTS methods. The DPPH method is based on the scavenging capacity of the essential oil against the radical 2,2-diphenyl-1-picrylhydrazyl (DPPH^•^) and in the ABTS method scavenging capacity was determined against the radical ion 2,2′-azino-bis(3-ethylbenzothiazoline-6-sulfonic acid) (ABTS^•+^). Table 5 shows the scavenging capacity (SC_50_) in µg/mL of the essential oil and the positive control (trolox), as well as the standard deviation (SD). The maximum evaluated concentration was 1000 µg/mL.

### 2.7. Anticholinesterase Activity

**Figure 1:**
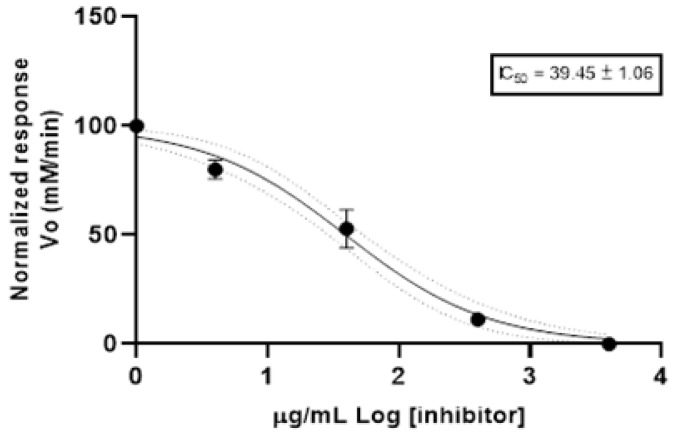
Anticholinesterase activity of essential oil from *Bixa orellana*.

The anticholinesterase (anti-AChE) activity was determined using the spectrophotometric method. Figure 1 shows the Log of concentration of EO and normalized response rate of reaction of acetylcholinesterase. The results are reported as half-maximal inhibitory concentration (IC_50_) value. *Bixa orellana* EO reported an IC_50_ value of 39.45 ± 1.06 µg/mL. The positive control (donepezil) exhibited an IC_50_ value of 12.40 ± 1.35 µg/mL.

## 3. Discussion

This article presents the study of the essential oil from *B. orellana* leaves obtained by hydrodistillation. The extraction yield was 1.3 ± 0.1 mL/kg (moisture of 64%), which could be considered low, since according to the categorization proposed by the “Science and Technology for Development” (CYTED), for plant species, the yield values in EO less than 5 mL/kg are considered low, values between 5 mL/kg and 10 mL/kg intermediate and values greater than 10 mL/kg high [23]. Caixeta et al. in 2020 determined that the yield in the EO of dry leaves (moisture of 10.6%) from B. orellana was 0.21% (2.1 mL/kg) [24]. The potential application of essential oils should be evaluated from a quantitative and qualitative point of view. From a bioeconomy perspective, the biodiversity has limitations of production, and different efforts have been applied to improve extraction yields and make it sustainable. Some strategies include the use of co-products or by-products of the industrialization of primary cultivars; this could be the case of industrialization of annatto as a food additive where the isolation of other natural active principles from the leaves could be implemented for obtaining essential oils. The EO of *B. orellana* was characterized by its physical properties; the density and refractive index are useful measurements to declare the purity, quality and chemical composition [25], and it is known that these properties vary with the composition and represent the average weighting.

According to previous reports of the chemical composition of the EO of *B. orellana* leaves, Lawrence et al. in 1973 reported ishwarane (54%) a sesquiterpene hydrocarbon (C_15_H_24_) as the main compound [26], while Zollo et al. in 1998 identified ishwarane (43.5%), guaiol (7.3%) and γ-murolene (6.2%) [27]. More recently, Giwa-Ajeniya et al. in 2016 identified twenty-one volatile compounds for *B. orellana* leaves from Nigeria, where the main compounds were SH α-guaiene (49.3%, CF: C_15_H_24_), guaiol (8.1%), valencene (7.7%) and β-elemene (5.9%) [28]. Oliveira et al. in 2020 reported isocaryophyllene (26.58%, CF: C_15_H_24_), 1R-α-pinene (19.49%) and β-pinene (12.04%) as the main components for fresh *B. orellana* leaves collected in Brazil [20]. Caixeta et al. in 2020 determined 21 chemical constituents, which represent 75% of the total composition, in the EO of the B. orellana dry leaves collected in Brazil; the main compounds were SH α-humulene (43.01%), OS E-nerolidol (14.40%) and spathulenol (7.57%) [24]. The chemical composition of the volatile fraction of *B. orellana* leaves also has been studied for other extraction methods. Raga et al. in 2011 reported the chemical composition of a dichloromethane extract from Philippines and identified the presence of ishwarene, phytol, polyprenol and mixture of stigmasterol and sitosterol [29]. In another study, Giorgi et al. in 2013 used the headspace solid-phase microextraction method to isolate the volatile compounds of *B. orellana* leaves from Brazil and reported 𝛽-caryophyllene (22.39%), 𝛾-elemene (12.45%), 𝛼-caryophyllene (12.00%), 𝛼-copaene (11.44%) and D-germacrene (10.09%) [11].

Ishwarane, a bioactive sesquiterpene hydrocarbon, has not been identified in our study, even as traces; however, it was reported by Lawrence et al. in 1973 [26], Zollo et al. in 1998 [27] and Raga et al. in 2011 [29], and in the essential oil of *B. orellana* seeds. Pino and Correa in 2003 presented the composition of EO of *B. orellana* seeds, from the 35 components identified the main were (Z,E)-farnesyl acetate (11.6%), occidentalol acetate (9.7%), spathulenol (9.6%) and ishwarane (9.1%) [21]. In the same way, Monzote et al. in 2013 reported ishwarane (18.6%) and geranylgeraniol (9.1%) as major compounds of *B. orellana* seeds [12]. The chemical composition of the EO of *B. orellana* of the present study was majorly grouped as sesquiterpene hydrocarbons (69.06%), similar to the report of Giorgi et al. in 2013 [11]. The chemical composition of EO of *B. orellana* leaves presents differences with the compared references. This is a fact that could be accounted for by the variations in cultivar practices, since compounds present in an EO depend on intrinsic factors (plant part, plant age, phenological state, etc.) and extrinsic factors (soil type, shade, amount of rainfall, etc.) of the plants. This species is cultivated in Central and South America and other tropical countries around the world where there are different climatic conditions and types of soil [17].

The enantioselective GC-MS analysis performed in this study is the first report for the EO of *B. orellana*, which showed six chiral compounds as presented in Table 3. The (−)-α-pinene and (+)-β-pinene were presented as pure (e.e > 90%) along with another four mixtures: (+/−)-α-copaene (e.e 82.04%), (+/−)-α-trans-bergamotene (e.e 61.88%), (+/−)-bicyclogermacrene (e.e 82.09%) and (+/−)-spathulenol (e.e 69.61%). The identification of chiral compounds in natural products is related with the bioactive properties and the bioavailability; in EO they are also related to sensorial properties, quality and purity [30].

Regarding to the antimicrobial activity Van Vuuren and Holl in 2017 [31] recommended a scale for MIC to recognize the potency of natural extracts. They proposed for essential oils that values of MIC > 1001 µg/mL are considered inactive, MIC between 500 and 1000 µg/mL have moderate activity, MIC between 101 and 500 µg/mL have strong activity and MIC less than 100 µg/mL have very strong activity. When applying this scale to the EO of *B. orellana* leaves, it showed strong activity (MIC 250 µg/mL) against *Enterococcus faecium* (ATCC 27270), moderate activity (MIC 1000 µg/mL) against *Enterococcus faecalis* (ATCC 19433) and *Staphylococcus aureus* (ATCC 25923), while it was inactive (MIC > 1001 µg/mL) against Gram-positive bacilli and Gram-negative bacilli. The positive control shows MIC values of around 1 µg/mL, so it should be considered that the positive control is a pure synthetic substance while the EO is a mixture of natural compounds. Based on these results, EO of *B. orellana* leaves would have antimicrobial potential against three of the seven bacteria tested. Coelho dos Santos et al. in 2022 presented a review of the antimicrobial activity reported for extracts of different parts of *B. orellana*; however, there are no data for essential oils of the leaves [32].

The antioxidant activity was reported as the SC_50_, the results showed moderate scavenging capacity for the DPPH assay (SC_50_ 224.24 ± 6.4 µg/mL) and strong capacity for the ABTS assay (SC_50_ 61.49 ± 0.04 µg/mL) the differences could be related to the reaction mechanism of each antioxidant method, because in DPPH assay terpene compounds are less capable of donating a hydrogen atom [33]. The antioxidant activity has been related to the possible applications of the EO, and generally this activity responds to synergistic or antagonistic effects among the chemical components present in the mixture. As a comparison of the antioxidant activity, we cited Shilpi et al. (2006); they evaluated the methanol extract of *B. orellana* L. leaves and reported an IC_50_ 22.36 μg/mL in the DPPH assay and antibacterial activity against selected causative agents of diarrhea and dysentery, including *Shigella dysenteriae* [17].

Cholinesterase is a term referring to one of the two enzymes, named, acetylcholinesterase and pseudocholinesterase. Both compounds catalyze the hydrolysis of excess neurotransmitter acetylcholine, and the excessive activation caused by acetylcholine would produce damage to the neuron or muscle. A cholinesterase inhibitor is known as an anticholinesterase compound [34]. Anticholinesterase compounds are also used for treating myasthenia gravis, glaucoma and Alzheimer’s disease. Synthetic anticholinesterase compounds are potent neurotoxins of moderate effectiveness, of high cost and short half-life that can also cause gastrointestinal disturbances, so currently compounds isolated from natural products (plants) are increasingly being explored for their anticholinesterase properties and their better secondary effects [35]. Therefore, different studies report the anticholinesterase activity of essential oils considering the potential uses in the treatment of Alzheimer’s disease [36]. This is the first report of anticholinesterase activity for the EO of *B. orellana*; the IC_50_ was 39.45 ± 1.06 μg/mL, this value could be considered as moderate potency when it is compared with the scale proposed by Santos et al. (2018) [35], which values high potency IC_50_ < 20 µg/mL; moderate potency 20 < IC_50_ < 200 µg/mL; and low potency 200 < IC_50_ < 1000.

## 4. Materials and Methods

### 4.1. Materials

Helium was purchased from INDURA (Quito, Ecuador). Mueller-Hinton broth, Mueller-Hinton II broth and fluid thioglycolate medium were purchased from DIPCO (Quito, Ecuador). The standard aliphatic hydrocarbons were purchased from ChemService (West Chester, PA, USA). Acetylcholinesterase (AChE), acetylthiocholine (AcSCh), dichloromethane (DMC), dimethyl sulfoxide (DMSO), methanol (MeOH), 2,2-diphenyl-1-picrylhydryl (DPPH), 2,2′-azinobis-3-ethylbenzothiazoline-6-sulfonic acid (ABTS), 5,5′-dithiobis (2-nitrobenzoic acid) (DTNB), butylated hydroxytoluene (BHT), donepezil, magnesium chloride hexahydrate, phosphate-buffered saline (PBS), sodium sulfate anhydrous, Trolox and tris hydrochloride (Tris-HCl) were purchased from Sigma-Aldrich (San Luis, MO, USA). All chemicals were of analytical grade and used without further purification.

### 4.2. Plant Material

The leaves of *B. orellana* were collected in the surroundings of the El Dorado Parish, Francisco de Orellana Canton, Orellana Province. The collection was carried out in the place that is located at 0°29′48” south longitude and 76°54′41” west latitude and an altitude of 225 m.a.s.l. After being collected, the plant material was stored and transferred in airtight plastic containers.

### 4.3. Essential Oil Isolation

A Clevenger type apparatus was used for the isolation of essential oil, in which the extraction of the oil was carried out by hydrodistillation. An 80 L distiller was used, in which approximately 18 L of water was placed. The process was maintained for 3 h counted from the fall of the first drop of distillate. The condensed essential oil was separated from the water by decantation, then the essential oil was dried using anhydrous sodium sulfate and stored at 4 °C in amber sealed vials until it was used in analysis.

### 4.4. Determination of the Physical Properties of the Essential Oil

Density of the EO was determined using the ISO 279:1998 standard (equivalent to the AFNOR NF T 75–111 standard) using an analytical balance (Mettler AC 100, Mettler Toledo, Columbus, OH, USA) and a pycnometer of 1 mL. Refractive index was determined using the standard ISO 280:1998 (similarly to AFNOR NF T 75–112) using a refractometer (model ABBE, BOECO, Hamburg, Germany). Optical rotation of the EO was determined according to the standard ISO 592:1998 using an automatic polarimeter (Mrc-P810, MRC, Holon, Israel). The subjective color was obtained online, for which a photograph of the EO taken with a white background was uploaded to the PINETOOL website (https://pinetools.com/, accessed on 20 July 2022). All measurements were taken at 20 °C.

### 4.5. Identification and Quantification of Essential Oil Compounds

The analysis of chemical composition was carried out in a gas chromatograph (GC) (model 6890N series, Agilent Technologies, Santa Clara, CA, USA). For qualitative analysis, the GC was coupled to a quadrupole mass spectrometer (MS) (model Agilent series 5973 inert, Agilent Technologies, Santa Clara, CA, USA) and for quantitative analysis the GC was equipped with a flame ionization detector (FID). In both cases, a nonpolar chromatographic column (Agilent J&W DB-5ms Ultra Inert GC column, Agilent Technologies, Santa Clara, CA, USA) with stationary phase 5%-phenyl-methylpolyxilosane, which was 30 m long, 0.25 mm of internal diameter and 0.25 µm of stationary phase thickness was used. The GC was equipped with a split/splitless autosampler (model 7683, Agilent Technologies, Santa Clara, CA, USA). The supply of hydrogen for the FID was carried out using a gas generator (model 9150, Packard, Conroe, TX, USA). The EO sample was prepared at 1% (*v*/*v*) by putting 10 μL of EO and 990 μL of dichloromethane in an amber vial. For the qualitative and quantitative analyses, a 1 μL of sample was injected in split mode with a partition ratio of 40:1, at a temperature of 220 °C and a pressure of 11 psi. In both cases, the chromatographic run began maintaining the initial temperature of 50 °C for 3 min, then the temperature was increased by 3 °C/min until reaching the final temperature of 230 °C, which was maintained for 3 min. For GC-MS, a constant flow of helium was maintained at a rate of 0.9 mL/min and a velocity of 23 cm/s, and for GC-FID the flow was 1.0 mL/min and the speed was 40 cm/s. Equation 1 [37] was used to determine the retention index (RI) of each compound. For the identification of the compounds, the IR and the mass spectra were compared with those existing in the bibliography [38,39].

(1) $$\mathrm{RI}=100C+100\frac{\left(\mathrm{RTx}-\mathrm{RTn}\right)}{\left(\mathrm{RTN}-\mathrm{RTn}\right)}$$

where C is carbon number of aliphatic hydrocarbons (C_9_ to C_25_) that elute before the compound of interest, RTx is the retention time of the compound of interest, RTn is the retention time of aliphatic hydrocarbons that elute before the compound of interest and RTN is the retention time of hydrocarbons that elute after the compound of interest.

### 4.6. Enantioselective Analysis

For enantiomeric analysis, gas chromatography (Trace 1310, Thermo Fisher Scientific, Waltham, MA, USA) coupled to mass spectrometry (quadrupole) (ISQ 7000, Thermo Fisher Scientific, Waltham, MA, USA) was used. Analyses were performed on an enantioselective GC column (MEGA-DEX DMT-Beta, Mega, Legnano, MI, Italy) of 30 m length, 0.25 m internal diameter and 0.25 μm thick stationary phase (2.3-diethyl-6-tert-butyldimethylsilyl-β-cyclodextrin). Sample preparation, amount injected, injection temperature and partition radius were those described for GC-MS. The carrier gas used was helium with a flow of 1.0 mL/min and a speed of 40 cm/s. The chromatographic run began by maintaining the oven at 60 °C for 5 min, then the temperature was increased with a ramp of 2 °C/min up to 230 °C, finally this temperature was maintained for 5 min. The calculation of the enantiomeric excess and elution order was carried out according to the procedures previously described by Morocho et al. in 2023 [34].

### 4.7. Antimicrobial Activity

The antibacterial activity of the essential oil was tested against seven strains of bacteria: three Gram-positive cocci bacteria including *Enterococcus faecalis* (ATCC 19433), *Enterococcus faecium* (ATCC 27270) and *Staphylococcus aureus* (ATCC 25923); a Gram-positive bacilli bacterium *Listeria monocytogenes* ATCC 19115; and three Gram-negative bacilli bacteria including *Escherichia coli* O157:H7 (ATCC 43888), *Pseudomonas aeruginosa* (ATCC 10145) and *Salmonella enterica* subs enterica serovar Thypimurium WDCM 00031, derived (ATCC 14028). The broth microdilution method was used to determine this activity, the procedures were performed as previously described by Valarezo et al. in 2021 [40]. The maximum evaluated concentration was 4000 µg/mL. Ampicillin and ciprofloxacin were used as a positive control and DMSO was used as a negative control.

### 4.8. Evaluation of Antioxidant Capacity

The DPPH and ABTS methods were used to determine free radical scavenging activity of EO from *B. orellana*. The antioxidant capacity of EO was determined according to the procedure described by Salinas et al. [41], using a UV spectrophotometer (Genesys 10S UV-Vis Spectrophotometer, Thermo Fisher Scientific, Waltham, MA, USA). In the DPPH method, 2,2-diphenyl-1-picrylhydrazyl radical was produced from the reagent 2,2-diphenyl-1-picrylhydrazyl and the absorbance of the samples was measured at a wavelength of 515 nm. In the ABTS method, 2,2′-azinobis (3-ethylbenzothiazoline-6-sulfonic acid) radical cation was produced from reagent 2,2′-azinobis (3-ethylbenzothiazoline-6-sulfonic acid) and the measurement of the absorbance of the samples was carried out at a wavelength of 734 nm. The SC_50_, which is the concentration value necessary for the EO to have a scavenging capacity of half of the radicals, was used to express the antioxidant activity. Trolox and methanol were used as a positive and negative control, respectively.

### 4.9. Anticholinesterase Activity

The spectrophotometric method was used to determine the acetylcholinesterase inhibitory effect of the EO of leaves from *B. orellana*. The procedures were performed as previously described by Valarezo et al. [42]. Measurements were made in a microplate spectrophotometer (EPOCH 2, BioTek, Winooski, VT, USA) at a wavelength of 405 nm. The IC50 was used to express the anticholinesterase activity. IC50 is the concentration of EO required for 50% inhibition. Methanol and donepezil hydrochloride were used as a negative and positive control, respectively.

### 4.10. Statistical Analysis

All procedures were performed in triplicate, except the identification of essential oil compounds, enantioselective analysis and antimicrobial activity, which were performed nine times. The data were collected in a Microsoft Excel sheet. The statistical software Minitab 17 (Version 17.1.0., Minitab LLC., State College, PA, USA) was used to calculate the measures of central tendency and standard deviation.

## 5. Conclusions

The enantiomeric distribution, antimicrobial activity, antioxidant capacity and anticholinesterase activity of essential oil from leaves of *Bixa orellana* were determined for the first time. Fifty-six chemical compounds and six pairs of enantiomers were identified in the essential oil. The main compound was germacrene D. Essential oil exhibited strong antibacterial activity against one of the tested strains, making this oil suitable for formulations in the pharmaceutical industry of specific antimicrobial products for this type of bacterial strain. Furthermore, essential oil presented a strong antioxidant activity and moderate anticholinesterase activity, which makes it novel for the food industry where it could be used to formulate functional foods. With this research, new information is provided on the species of aromatic plants of Ecuador, thus contributing to the knowledge of Ecuadorian biodiversity. For future studies it is proposed to test the anti-inflammatory or anti-repellent activity.

## Figures and Tables

**Table 1:** Physical properties of the essential oil of *Bixa orellana*.

Property	*Bixa orellana* EO	
	Mean	SD
Density, ρ (g/cm^3^)	0.8884	0.0063
Refractive index, *n*^20^	1.4714	0.0145
Specific rotation, [α] (°)	+12.48	0.02
Subjective color	Yellow	
RGB color values	R:240, G:224, B:36	
CMYK color values	C:0, M:7, Y:85, K:6	
Hex Color Codes	#f0e024	

**Table 2:** Chemical composition of essential oil from the leaves of *Bixa orellana*.

CN	RT	Compound	RIC	RIR	%	SD	Type	CF	MM (Da)
1	7.11	Pinene <α->	933	932	6.34	0.13	MH	C_10_H_16_	136.13
2	9.16	Pinene <β->	975	974	4.11	0.87	MH	C_10_H_16_	136.13
3	9.69	Myrcene	989	988	0.65	0.04	MH	C_10_H_16_	136.13
4	11.89	Phellandrene <β->	1026	1025	0.15	0.05	MH	C_10_H_16_	136.13
5	12.01	Sylvestrene	1027	1025	0.40	0.01	MH	C_10_H_16_	136.13
6	12.29	Ocimene <(Z)-β->	1033	1032	2.32	0.19	MH	C_10_H_16_	136.13
7	13.62	Terpinene <γ->	1056	1054	0.37	0.11	MH	C_10_H_16_	136.13
8	30.73	Elemene <δ->	1335	1335	0.55	0.09	SH	C_15_H_24_	204.19
9	31.35	Cubebene <α->	1347	1348	0.28	0.08	SH	C_15_H_24_	204.19
10	32.84	Copaene <α->	1376	1374	1.94	0.50	SH	C_15_H_24_	204.19
11	33.49	Cubebene <β->	1388	1387	0.11	0.04	SH	C_15_H_24_	204.19
12	33.60	Elemene <β->	1390	1389	1.18	0.08	SH	C_15_H_24_	204.19
13	34.58	Cedrene <α->	1410	1410	0.10	0.02	SH	C_15_H_24_	204.19
14	34.75	Bergamotene <α-cis->	1413	1411	0.43	0.10	SH	C_15_H_24_	204.19
15	35.04	Caryophyllene <(E)->	1419	1417	8.56	1.24	SH	C_15_H_24_	204.19
16	35.66	Bergamotene <α-trans->	1434	1432	0.73	0.14	SH	C_15_H_24_	204.19
17	35.92	Aromadendrene	1439	1439	0.82	0.02	SH	C_15_H_24_	204.19
18	36.28	Himachalene <α->	1447	1449	0.41	0.03	SH	C_15_H_24_	204.19
19	36.43	Muurola-3,5-diene <trans->	1451	1451	0.14	0.02	SH	C_15_H_24_	204.19
20	36.74	Humulene <α->	1456	1452	1.23	0.30	SH	C_15_H_24_	204.19
21	36.92	Santalene <β->	1461	1457	0.67	0.04	SH	C_15_H_24_	204.19
22	37.37	Aristolochene <4,5-di-epi->	1471	1471	3.18	0.03	SH	C_15_H_24_	204.19
23	37.52	Cadina-1(6),4-diene <trans->	1474	1475	0.42	0.06	SH	C_15_H_24_	204.19
24	37.69	Muurolene <γ->	1478	1478	0.60	0.02	SH	C_15_H_24_	204.19
25	37.97	Germacrene D	1483	1480	17.87	1.20	SH	C_15_H_24_	204.19
26	38.07	Himachalene <γ->	1486	1481	0.78	0.15	SH	C_15_H_24_	204.19
27	38.20	Selinene <δ->	1489	1492	0.63	0.12	SH	C_15_H_24_	204.19
28	38.36	Viridiflorene	1493	1496	2.44	0.22	SH	C_15_H_24_	204.19
29	38.45	Valencene	1495	1496	0.91	0.10	SH	C_15_H_24_	204.19
30	38.62	Bicyclogermacrene	1499	1500	14.27	0.97	SH	C_15_H_24_	204.19
31	38.75	Muurolene <α->	1502	1500	0.83	0.04	SH	C_15_H_24_	204.19
32	38.90	Epizonarene	1505	1501	0.29	0.06	SH	C_15_H_24_	204.19
33	39.00	Farnesene <(E,E)-α->	1507	1505	2.60	0.18	SH	C_15_H_24_	204.19
34	39.12	Bisabolene <(Z)-α->	1509	1506	0.54	0.09	SH	C_15_H_24_	204.19
35	39.39	Cadinene <γ->	1516	1513	0.85	0.24	SH	C_15_H_24_	204.19
36	39.60	Cadinene <δ->	1522	1522	4.98	0.58	SH	C_15_H_24_	204.19
37	39.82	Calamenene <cis->	1528	1528	0.49	0.08	SH	C_15_H_22_	202.17
38	40.12	Liguloxide	1535	1535	tr	-	OS	C_15_H_26_O	222.20
39	40.23	Cadinene <α->	1537	1537	0.23	0.04	SH	C_15_H_24_	204.19
40	41.45	Longipinanol	1567	1567	3.04	0.47	OS	C_15_H_26_O	222.20
41	42.19	Spathulenol	1582	1577	2.74	0.45	OS	C_15_H_24_O	220.18
42	42.51	Thujopsan-2-β-ol	1593	1588	1.12	0.16	OS	C_15_H_26_O	222.20
43	42.85	Viridiflorol	1598	1592	0.32	0.08	OS	C_15_H_26_O	222.20
44	42.93	Guaiol	1604	1600	0.41	0.15	OS	C_15_H_26_O	222.20
45	43.43	Junenol	1619	1618	0.68	0.03	OS	C_15_H_26_O	222.20
46	44.04	Acorenol <α->	1638	1632	0.20	0.01	OS	C_15_H_26_O	222.20
47	44.14	Cadin-4-en-7-ol <cis->	1640	1635	0.36	0.03	OS	C_15_H_26_O	222.20
48	44.26	Acorenol <β->	1641	1636	0.84	0.09	OS	C_15_H_26_O	222.20
49	44.48	Agarospirol	1651	1646	0.05	0.00	OS	C_15_H_26_O	222.20
50	44.57	Himachalol	1654	1652	0.44	0.11	OS	C_15_H_26_O	222.20
51	44.65	Cadinol <α->	1656	1652	1.84	0.48	OS	C_15_H_26_O	222.20
52	45.02	Intermedeol <neo->	1664	1658	1.24	0.10	OS	C_15_H_26_O	222.20
53	45.15	Intermedeol	1669	1665	0.14	0.01	OS	C_15_H_26_O	222.20
54	45.57	Cedranol <5-neo->	1684	1684	1.63	0.30	OS	C_15_H_26_O	222.20
55	45.68	Germacrone	1688	1693	0.54	0.03	OS	C_15_H_22_O	218.17
56	52.72	Bifloratriene	1982	1977	0.26	0.04	DH	C_20_H_32_	272.25
		Monoterpene hydrocarbons			14.34				
		Sesquiterpene hydrocarbons			69.06				
		Oxygenated sesquiterpene			15.59				
		Diterpene hydrocarbons			0.26				
		Total identified			99.25				

CN: compound number; RT: retention time; RIC: calculated retention indices; RIR: reference retention indices; %: relative abundance; CF: chemical formula; MM: monoisotopic mass; SD: standard deviation; Tr: traces.

**Table 3:** Chiral compounds present in the essential oil of the leaves from *Bixa orellana*.

RT	Enantiomers	RI	ED (%)	e.e. (%)
4.12	(+)-α-Pinene	935	0.19	99.63
4.25	(−)-α-Pinene	940	99.81	
5.82	(+)-β-Pinene	997	95.60	91.20
6.07	(−)-β-Pinene	1004	4.40	
26.81	(+/−)-α-Copaene	1375	8.98	82.04
27.04		1379	91.02	
30.06	(+/−)-α-trans-Bergamotene	1429	19.06	61.88
30.28		1433	80.94	
35.49	(+/−)-Bicyclogermacrene	1522	91.05	82.09
35.73		1526	8.95	
43.52	(+/−)-Spathulenol	1665	15.20	69.61
43.77		1670	84.80	

**Table 4:** Antibacterial activity of essential oil from *Bixa orellana*.

Microorganism	Essential oil	Positive control	Negative control
	MIC (µg/mL)		
**Gram-positive cocci**			
*Enterococcus faecalis* (ATCC 19433)	1000	0.78	+
*Enterococcus faecium* (ATCC 27270)	250	0.39	+
*Staphylococcus aureus* (ATCC 25923)	1000	0.39	+
**Gram-positive bacilli**			
*Listeria monocytogenes* ATCC 19115	2000	1.56	+
**Gram-negative bacilli**			
*Escherichia coli* O157:H7 (ATCC 43888)	>4000	1.56	+
*Pseudomonas aeruginosa* (ATCC 10145)	>4000	0.39	+
*Salmonella enterica* subs enterica serovar Thypimurium WDCM 00031, derived (ATCC 14028)	>4000	0.39	+

+: normal growth.

**Table 5:** Antioxidant activity of essential oil from *Bixa orellana*.

Sample	DPPH	ABTS
	SC_50_ (µg/mL) ± SD	
*Bixa orellana* essential oil	224.24 ± 6,4	61.49 ± 0.04
Trolox	29.99 ± 1.1	23.27 ± 1.1

## Data Availability

Data are available from the authors upon reasonable request.
